# Supplementary material for: Simulation-guided engineering of split GFPs with efficient β-strand photodissociation
Source: Nat Commun. 2023 Nov 16;14:7401. doi: 10.1038/s41467-023-42954-4 (PMC10654500; doi:10.1038/s41467-023-42954-4)
Supplement: Supplementary file 1 — Supplementary Information [file 41467_2023_42954_MOESM1_ESM.pdf]

Supplementary Information for

Simulation-guided engineering of split GFPs with efficient β-strand photodissociation

Yasmin Shamsudin[1,2], Alice R. Walker[1,3], Chey M. Jones[1], Todd J. Martínez[1] & Steven G. Boxer[1]**

Correspondence to: sboxer@stanford.edu and yasmin.shamsudin@kemi.uu.se

This PDF file includes:

Computational methods
Experimental materials and methods
Supplementary Text
Supplementary Figs. 1 to 6
Supplementary Tables 1 to 4

Table of Contents

Computational Methods 4

S1. Modelling the split GFP complex 4

S2. Molecular dynamics simulations 5

S3. Consensus structure modelling 5

S4. Modelling mutants 5

S5. Umbrella sampling 6

S6. QM/MM methods 6

S7. Hydrogen bond analysis 7

S8. Simulated annealing simulations 8

Experimental Materials and Methods 9

Note that the superfolder GFP nomenclature for Tyr200, Ser202, Lys209, and Asn121 correspond to Tyr10, Ser12, Lys19, and Asn183, respectively, in the computational models, in accordance with the 6OFO crystal structure (see Supplementary Fig. 5 or Supplementary Table 3). 9

S9. DNA sequences 9

S10. Mutations 9

S11. Amino acid sequences 10

S12. Experimental mutagenesis 11

S13. Protein expression 11

S14. Protein purification 11

S15. Protein cleavage 12

S16. Mass spectrometry 12

S17. UV-Vis absorbance measurements 12

S18. Laser-dissociation experiments 13

S19. Fluorescence measurements 13

Supplementary text 14

S20. Electronic states 14

S21. Umbrella sampling simulations 15

S21. Rotational correlation analysis and hydrogen bond occupancies 16

S22. Mutations of Asn121Ala 16

Supplementary Fig. 1 RMSD of replicated *cis* and *trans* simulations 18

Supplementary Fig. 2 Electronic states 19

Supplementary Fig. 3. Hydrogen bond networks in the *cis* and *trans* complexes 20

Supplementary Fig. 4. PMF plots 21

Supplementary Fig. 5. Schematic topology of split GFP 22
Supplementary Fig. 6 Electronic state methods 23
Supplementary Table 1. Strand exchange rates 24
Supplementary Table 2. Experimental fluorescence quantum yields 24
Supplementary Table 3. Conversion table between computational models and sfGFP crystal structure 25
Supplementary Table 4. Mass spectrometry 26

Computational Methods

S1. Modelling the split GFP complex

Our reference protein is a circularly permuted variant of the superfolder GFP (PDB ID: 2B3P)[14], with an added sacrificial loop for enzymatic cleavage and dissociation of β-strand 10 (see complete DNA and amino acid sequences in the SI). A closely homologous protein structure with the chromophore in the *cis* conformation has been determined (PDB ID: 6OFO)[9]. It contains two mutations (Cys48Ser and Cys70Ala), unresolved loops on both ends of strand 11, and multiple partially unresolved sidechains, mainly on loops and the solvent-exposed face of the barrel.[10] Thus, further modeling was required to obtain starting structures suitable for computational simulations, corresponding to the split reference protein. Therefore, the 6OFO[9] crystal structure was used as the template for homology modeling of the reference protein with both cysteines restored, and Glu222 in the protonated form. The two models generated using Modeller[15] and one model created using Prime[16] (see Fig. 2b) contained the β-barrel, internal α-helix, and the missing loops, excluding the sacrificial loop (see p. 10 in this SI for full sequence) and the chromophore, which was separately modelled (see below).

The *cis* and *trans* chromophores (Fig. 2d and S2) were built using the Build menu in Maestro (Bioluminate 4.3[17]) and minimized using MacroModel[17] before optimizing the structures with GAUSSIAN16[18] using B3LYP/6-31G(d). The obtained RESP charges were fitted with Antechamber.[19] The chromophores were manually merged with the internal α-helix of each homology model, and the charges at the interface were manually corrected to create systems with integer charges. Additional parameters were obtained from the GAFF2[19] and FF14SB[20] force fields.

Three complexes were created with the *cis* chromophore, and three models with the *trans* chromophore. We will refer to complexes containing the chromophore modeled in the *cis* conformation as *cis* complexes and the corresponding complexes with the chromophore modeled in the *trans* conformation as *trans* complexes.

To maintain consistency with previous works on GFP, we use the superfolder GFP (sfGFP) nomenclature in our discussions, despite differences in our complexes due to the circular permutation placing the N-terminal on strand 10 and added residues due to the sacrificial loop (see Supplementary Fig. 5 for comparison). However, since the cleavage site of the loops

between strands 10 and 11 do not have corresponding amino acids in sfGFP (see Supplementary Table 3 for conversion table), we will refer to the amino acids by their computational model numbers. Thus, the six complexes were manually cleaved between Arg26(comp) and His27(comp) and protons were automatically added to the new terminals. The terminal sidechains were then selectively minimized using MacroModel[17] to prevent steric clashes between terminal protons in the subsequent simulations.

S2. Molecular dynamics simulations

For all simulations aiming to create starting consensus and mutant structures for isomerization or enhanced sampling simulations, the minimization, heating, and equilibration protocol was as follows: The complex was prepared using the Antechamber *tleap* module, which assigned protein parameters using the FF14SB and GAFF2 forcefields, and the complex was placed in an octahedral simulation box filled with SPC/E waters[21] and counterions. The starting structure was minimized, followed by heating to 300 K (NVT) over 250 ps, and 5 ns of equilibration (NPT). The *pmemd* module in AMBER 2018[22] was used for all simulations. The step size during heating and equilibration and unconstrained MD (NPT) simulations was 2 fs, using SHAKE and RATTLE constraints. The Langevin thermostat[23] was used, with a collision frequency of 2 ps^{-1}, and the cutoff for non-bonded interactions was set at 9 Å.

S3. Consensus structure modelling

Following equilibration, the proteins were subjected to three replicates of unconstrained 1 μs MD simulations. The trajectories of the final 5 ns for each simulation were manually inspected. The simulations were considered converged as the loops looked similar (average heteroatom RMSD: 3.6 Å) regardless of starting point. The consensus model was created by merging the barrel with representative loop regions extracted from the last frame of one simulation with each chromophore conformation.

S4. Modelling mutants

Point mutations of Tyr200, Ser202, Lys209, and Asn121 were introduced on the consensus *cis* structures using the Mutate Residue option in the Build menu in Maestro. Rotamers of bulky ligands were chosen to minimize steric clashes. The triple mutations AAA

(Tyr200Ala, Ser202Ala, and Lys209Ala) and IWI (Tyr200Ile, Ser202Trp, and Lys209Ile), and quadruple mutations with Asn121Ala, AAA-A and IWI-A, all with the chromophore modeled in the cis conformation, were computationally modelled. Following equilibration, the mutated structures were subjected to three replicates of 20 ns of unconstrained MD simulations at 300 K. Frames from these simulations were used as starting structures for umbrella sampling simulations (see below).

S5. Umbrella sampling

To investigate factors that promote or inhibit *cis–trans* isomerization of the chromophore in the superfolder strand 10 circular permutant, we used the *cis* consensus model as a basis for classical umbrella sampling on the ground state. The *cis* structures from the end of unconstrained, relaxed classical MD trajectories at 300 K were used as the starting structures for umbrella sampling simulations using the *pmemd.cuda* module in AMBER 2018[22] with classical molecular dynamics. Specifically, we use biased MD simulations to model *cis–trans* isomerization in split GFP by gradually rotating and sampling the chromophore along its ϕ_I coordinate. Because the protein environment surrounding the chromophore is asymmetric, we generated potential of mean force (PMF) profiles associated with clockwise and counter-clockwise rotations along ϕ_I (Fig. 2d). The ϕ_I dihedral angle of the chromophore was rotated in increments of 10° in both directions using a harmonic constraint of 200 kcal/mol. Each window began with the final snapshot of the previous window, equilibrated for 50 ps, and run for 1 ns in the NPT ensemble with a 1 fs timestep. The classical sampling was performed with Langevin thermo/barostat with a collision frequency of 1 ps^{-1}. The resulting PMFs (Supplementary Fig. 4) were constructed using the weighted histogram analysis method (WHAM).

S6. QM/MM methods

The QM/MM calculations were performed with the TeraChem 1.9.3/OpenMM7 interface.[24–29] The QM region was defined as the chromophore and adjacent capping residues (Supplementary Fig. 6a) while the MM region was the remaining protein and solvent. As justified in previous gas-phase and solution-phase studies of the anionic GFP chromophore,[11,12] the α-corrected implementation of the state-averaged complete active space self-consistent field (SA-CASSCF) method (known as α-CASSCF) was used to obtain optimized structures on the

ground (S_0) and excited (S_1) electronic states.[11,12] As illustrated in Supplementary Fig. 6b, we use an active space of four electrons and three orbitals, optimizing the orbitals to minimize the average energy of the first three singlet states, i.e. SA3-CAS(4/3)SCF. Following our previous work[11,12], α(0.64)-SA3-CASSCF(4,3)/6-31G* with a D3 dispersion correction was used in our split GFP system.

Optimizations for each electronic state were initiated from the same starting structures, which were sampled from classical umbrella sampling along ϕ_I. The ϕ_I dihedral angle was constrained for each optimization. Structures were optimized with QM/MM using the α(0.64)-SA3-CASSCF(4,3)/6-31G* level of theory. Explicit water molecules more than 10 Å from the chromophore were frozen in the optimizations. Geometries were obtained using GPU-accelerated TeraChem[28] and the DL-FIND[25] optimization library. These geometries were used to validate the choice of using the one-bond-flip mechanism, which is a rotation around the ϕ_I dihedral angle, while leaving the ϕ_P dihedral angle free or unchanging (Fig. 2d) and to determine sections of the umbrella sampling that were the most distorted compared to the excited state charges and geometry.

Using geometries sampled from the classical umbrella sampling, the potential energy surface of split GFP was mapped along ϕ_I (10° intervals) using QM/MM optimizations at the α(0.64)-SA3-CASSCF(4,3)/6-31G* level. S_0-optimized structures were obtained by constraining ϕ_I and allowing the remaining degrees of freedom of chromophore and protein to relax (Supplementary Fig. 2b). This method was benchmarked against extended multistate multireference second-order perturbation theory (XMS-CASPT2). Single point calculations at the SA3-XMS-CASPT2(4,3)/6-31G* level were run on α(0.64)-SA3-CASSCF(4,3)/6-31G* optimized geometries to ensure the reliability of the α parameter (Supplementary Fig. 6c).

S7. Hydrogen bond analysis

To limit the search space for potential mutagenesis, we first determined residues that had broken hydrogen bonds at least 30% of the simulation time in the umbrella sampling after moving the chromophore from *cis* to *trans* (Supplementary Fig. 3). Hydrogen bond analysis was performed with AMBER's cpptraj module. The standard cutoffs of 3 Å / 135° were used to compute hydrogen bond occupancy times for each possible protein atom. Residues that changed in hydrogen bond occupancy by ± 50% between the clockwise or counterclockwise rotation from

ϕ_I= 0 to 90° and 90° to 180° were extracted as a metric of residues that substantially change during isomerization.

S8. Simulated annealing simulations

Equilibrated consensus *cis* and *trans* structures were subjected to three replicates of enhanced sampling simulations using the *pmemd* module of AMBER as follows: the proteins were heated from 300K to 400 K over 1 ns, simulated at 400 K for 4 ns, heated to 600 K over 1 ns, simulated at 600 K for 4 ns, then cooled to 500 K over 1 ns before finally being simulated at 500 K for up to 150 ns. The trajectories were analyzed using VMD.

Experimental Materials and Methods

Note that the superfolder GFP nomenclature for Tyr200, Ser202, Lys209, and Asn121 correspond to Tyr10, Ser12, Lys19, and Asn183, respectively, in the computational models, in accordance with the 6OFO crystal structure (see Supplementary Fig. 5 or Supplementary Table 3).

S9. DNA sequences

Complete sequence for the reference structure, which is also referred to as YSK according to the mutation sites Tyr200 (Y), Ser202 (S), Lys209 (K). Codons for these sites, and Asn121 are highlighted in grey. The (TAA) ochre stop codon was used:

```
ATGGGCAGCAGCCATCATCATCATCATCACAGCAGCGGCCTGGTGCCGCGCGGCAGCCATATGC
TGCCGGATAACCATTATCTGAGCACCCAGACCGTGCTGAGCAAAGATCCGAACGAAGGCACCCG
CGGCAGCGGCAGCATTGAAGGCCGCCATAGCGGCAGCGGCAGCAAACGCGATCACATGGTGCTG
CATGAATATGTGAACGCGGCGGGCATTACCCATGGCATGGATGAACTGTATGGCGGCACCGGCG
GCAGCGCGAGCCAGGGCGAAGAACTGTTTACCGGCGTGGTGCCGATTCTGGTGGAACTGGATGG
CGATGTGAACGGCCATAAATTTAGCGTGCGCGGCGAAGGCGAAGGCGATGCGACCATTGGCAAA
CTGACCCTGAAATTTATTTGCACCACCGGCAAACTGCCGGTGCCGTGGCCGACCCTGGTGACCA
CCCTGAGCTATGGCGTGCAGTGCTTTAGCCGCTATCCGGATCACATGAAACGCCATGATTTTTT
TAAAAGCGCGATGCCGGAAGGCTATGTGCAGGAACGCACCATTAGCTTTAAAGATGATGGCAAA
TATAAAACCCGCGCGGTGGTGAAATTTGAAGGCGATACCCTGGTGAACCGCATTGAACTGAAAG
GCACCGATTTTAAAGAAGATGGCAACATTCTGGGCCATAAACTGGAATATAACTTTAACAGCCA
TAACGTGTATATTACCGCGGATAAACAGAAAAACGGCATTAAAGCGAACTTTACCGTGCGCCAT
AACGTGGAAGATGGCAGCGTGCAGCTGGCGGATCATTATCAGCAGAACACCCCGATTGGCGATG
GCCCGGTGCTGTAA
```

S10. Mutations

Codons used for single-point mutations.

**Residue**	**Codon**	**Residue**	**Codon**	**Residue**	**Codon**	**Residue**	**Codon**
Tyr200	TAT	Ser202	AGC	Lys209	AAA	Asn121	AAC
Ala200	GCT	Ala202	GCC	Ala209	GCA	Ala121	GCC
Ile200	ATT	Trp202	TGG	Ile209	ATT		
Leu200	CTT	Val202	GTC				
		Ile202	ATC				

S11. Amino acid sequences

Complete sequence for the reference structure, which is also referred to as YSK according to the mutation sites Tyr200 (Y), Ser202 (S), and Lys209 (K). The crystal structure sequence of superfolder GFP (sfGFP) and the quadruple mutant IWI-A included for comparison. Note that the strand 10 (green) is at the beginning of the sequence in YSK and IWI-A due to circular permutation, compared with sfGFP. The sequences are color-coded as follows: added peptide sequence of the pET-15b N-terminal His-tag (yellow), strand 10 (green), spacer loop (red), sacrificed loop (red italics), strand 11 (blue) chromophore (pink), and Cys (orange). The four mutation sites are highlighted in grey, with the trypsin protease cleavage sites indicated by ▼. The peptide exchanged for the dissociating β–strand, resulting in the formation of a yellow fluorescence protein (YFP), was designed to match the N-terminal β–strand 10 in our reference structure YSK (see below), except for the Thr203Tyr mutation, and was synthesized by Elim Biopharmaceuticals.

Ref (YSK):
MGSSHHHHHHSSGLVPR▼GSHMLPDNHYLSTQTVLSKDPNEGTR▼*GSGSIEGR*▼HSGSGSKRD
HMVLHEYVNAAGITHGMDELYGGTGGSASQGEELFTGVVPILVELDGDVNGHKFSVRGEGEGD
ATIGKLTLKFICTTGKLPVPWPTLVTTLSYGVQCFSRYPDHMKRHDFFKSAMPEGYVQERTISFK
DDGKYKTRAVVKFEGDTLVNRIELKGTDFKEDGNILGHKLEYNFNSHNVYITADKQKNGIKANF
TVRHNVEDGSVQLADHYQQNTPIGDGPVL

sfGFP:
MSKGEELFTGVVPILVELDGDVNGHKFSVRGEGEGDATNGKLTLKFICTTGKLPVPWPTLVTTLT
YGVQCFSRYPDHMKRHDFFKSAMPEGYVQERTISFKDDGTYKTRAEVKFEGDTLVNRIELKGID
FKEDGNILGHKLEYNFNSHNVYITADKQKNGIKANFKIRHNVEDGSVQLADHYQQNTPIGDGPV
LLPDNHYLSTQSVLSKDPNEKRDHMVLLEFVTAAGITHGMDELYK

IWI-A:
MGSSHHHHHHSSGLVPR▼GSHMLPDNHILWTQTVLSIDPNEGTR▼*GSGSIEGR*▼HSGSGSKRD
HMVLHEYVNAAGITHGMDELYGGTGGSASQGEELFTGVVPILVELDGDVNGHKFSVRGEGEGD
ATIGKLTLKFICTTGKLPVPWPTLVTTLSYGVQCFSRYPDHMKRHDFFKSAMPEGYVQERTISFK
DDGKYKTRAVVKFEGDTLVARIELKGTDFKEDGNILGHKLEYNFNSHNVYITADKQKNGIKANF
TVRHNVEDGSVQLADHYQXNTPIGDGPVL

Peptide sequence of the yellow (YFP) strand:
LPDNHYLSYQTVLSKDPNE

Peptide sequence of the reference (YSK) strand, highlighting the mutation sites and the Thr203 position (green).

LPDNHYLSTQTVLSKDPNE

S12. Experimental mutagenesis

The reference protein was obtained from gene synthesis (see p.9 in this SI for DNA sequence).[7] Point mutations were performed with the QuikChange Lightning Mutagenesis kit (Agilent) according to the manufacturer's protocol. Multiple point mutations were done in stages where Tyr200 and Ser202 were first mutated using one primer, followed by Lys209, and finally Asn121. The correct introduction of mutations was verified through DNA sequencing performed by ELIM Biopharm.

S13. Protein expression

The pET-15b vectors containing the genes of interest were transformed into chemically competent BL21(DE3) *Escherichia coli* (Invitrogen). Baffled 3-L flasks containing 1 L of 47.6 g/L modified Terrific Broth (Fisher BioReagents), 8 g/L glycerol (Fisher, CAS 56-81-5) and 100 mg/L ampicillin (Sigma-Aldrich, CAS 69-52-3) were inoculated with single colonies of *E. coli* and grown at 37ºC with shaking at 180 rpm until reaching OD 0.6 to 0.8 at 600 nm. Then, 0.25 g/L of isopropyl β-D-1-thiogalactopyranoside (Fisher, CAS 367-93-1) were added to the cultures to induce protein expression. The cultures were incubated for an additional 20 h at 20ºC while shaking at 180 rpm.

S14. Protein purification

E. coli containing the proteins of interest were pelleted by centrifugation at 6800 ×g (fixed angle, 6000 rpm) for 30 min at 4 °C. The cell pellets were suspended in lysis buffer, an aqueous buffer at pH 8.0 containing 50 mM Tris-HCl (Fisher, CAS 1185-53-1) and 250 mM NaCl (Fisher, CAS 7647-14-5). They were then lysed with a high-pressure homogenizer (Avestin EmulsiFlex-C3). The lysate was centrifuged at 26700 ×g (fixed angle, 15000 rpm) for 90 min at 4 °C. The resulting supernatant was added to a column of Ni-NTA Agarose resin (QIAGEN) pre-equilibrated with Buffer A (10m M NaCl, 20 mM Tris-HCl, pH 8.0 aqueous buffer). The column was rinsed with 2 column volumes of a wash buffer of 20 mM imidazole (Aldrich, CAS 288-32-4) in Buffer A before being rinsed with 2 column volumes of 200 mM imidazole in Buffer A. The fractions of eluate judged by visual inspection to contain GFP were pooled and exchanged by spin-filtration into anion-exchange Buffer A and stored at 4ºC overnight. The GFP was then purified by anion-exchange chromatography (HiTrap 5 mL Q HP;

GE Healthcare) with a gradient of Buffer A and B (1 M NaCl, 80 mM Tris-HCl, pH 8.0 aqueous buffer) and stored at 4ºC.

S15. Protein cleavage

Due to the light sensitivity, protein cleavage and subsequent purification of all mutants was done in the dark. The GFP was incubated at room temperature with 100 units of trypsin (Type III from bovine pancreas, ≥ 10,000 BAEE units per mg; Sigma) per 1 mg of GFP for 20 min while stirring constantly to cleave both the thrombin loops between the His-tags and the factor Xa loops after the N-terminal β-strands. The GFP was then purified by anion-exchange chromatography (HiTrap 5 mL Q HP; GE Healthcare) with a gradient of Buffers A and B (*vide supra*), followed by spin-filtration into anion-exchange Buffer A. Cleaved mutants were stored at 4ºC in anion-exchange Buffer A in the dark.

S16. Mass spectrometry

Mutant identities before and after cleavage were verified by electrospray ionization mass spectrometry (ESI-MS) measured with LC–MS (Waters 2795 HPLC with ZQ single quadrupole MS in Stanford University Mass Spectrometry (SUMS) facility. Deconvoluted masses were calculated using Intact Mass (Protein Metrics). Reported expected masses (Supplementary Table 4) are the average mass based on the sequence, calculated with the Peptide Mass Calculator provided by PeptideSynthetics.

S17. UV-Vis absorbance measurements

UV-Vis kinetic measurements were performed with a PerkinElmer Lambda 25 UV-Vis spectrometer. Data acquisition was performed every 1.0 nm at a maximum scan rate of 480 nm/min. UV-Vis measurements not for kinetic measurements were performed with a PerkinElmer Lambda 365 UV-Vis spectrometer. Mutant concentrations and extinction coefficients were determined by measuring the UV-Vis absorbance at 447 nm in 0.1 M NaOH (Fisher BioReagents, CAS 497-19-8) and scaling by the known extinction coefficient of the deprotonated chromophore in the denatured protein (44,100 M^{-1} cm^{-1})[30].

S18. Laser-dissociation experiments

A thermally conductive metal cuvette holder was affixed to a magnetic stir plate stirring at 1200 rpm. 4-mL quartz cuvettes with magnetic stir bars containing 3 μM of cleaved protein and 70 μM of the YFP peptide strand, with the single point T203Y mutation compared with our reference strand 10 (see peptide sequences on p. 10 in this SI), solvated in 3 mL Buffer A (*vide supra*) were incubated in the holder in the dark while stirring for 15 minutes before a control UV-vis measurement was taken. A 30 mW diode laser (85-BCD-030-115, Melles Griot) was used for irradiation with 488 nm light. Laser power was measured with a stabilized thermal power sensor (Part No. S302C, Thorlabs) coupled to a digital optical power console (Part No. PM100D, Thorlabs). The reported power (26 ± 1 mW) was scaled to 96% of the measured power due to 4% external reflection at the air-quartz interface on the surface of the cuvette containing the sample. It was then normalized based on sample volume. Sample irradiation was performed for defined time intervals using an automated shutter with millisecond precision (Model 845HP Digital Shutter, Newport Research). Upon completion of each irradiation time interval the sample was stirred continuously in the dark for 15 minutes before UV-vis measurement. Each mutant was subjected to three replicates of laser-dissociation experiments.

S19. Fluorescence measurements

Absolute fluorescence quantum yields were measured on uncleaved proteins by comparing GFP mutant fluorescence to fluorescence of the standard Fluorescein (Aldrich Chemicals, LOT 101F-0681, CAS 2321-07-5) in 0.1 M NaOH, which has a reported absolute fluorescence quantum yield of 0.90 at 488 nm.[31] Excitation was performed at 488 nm, and signal acquisition was performed every 0.5 nm at a scan speed of 120 nm/min with a slit width of 1.0 nm. Sample concentration was calibrated using a PerkinElmer Lambda 365 UV-Vis spectrometer. The fluorescence measurements were performed on a PerkinElmer LS 55 fluorescence spectrometer with emission and excitation slit widths of 2.5 nm.

Supplementary text

S20. Electronic states

In order to choose mutations to affect the isomerization pathway, we needed to both (1) validate that the classical umbrella sampling could approximate the protein's response to the chromophore along the path, and (2) that the particular reaction coordinate that we chose for the umbrella sampling led to a reasonable pathway on the excited state potential surface. We applied a combined quantum mechanics/molecular mechanics (QM/MM) partitioning scheme in order to describe electronic properties of split GFP on the ground and excited state surfaces, using a combination of the same forcefield parameters and protein setup as described in the molecular dynamics simulations section of the SI Methods and QM for the chromophore as described below. geometry was optimized from each window of the umbrella sampling twice, once on the ground state and once on the first excited state. In this way, we mapped out the potential energy surface that the classical umbrella sampling approximated, and found specific regions of difference between force field, QM/MM on the ground state, and QM/MM on the excited state. We found that the largest differences in charge and geometry were found near the conical intersection, but that generally the umbrella sampling gave a plausible approximation for the description of the chromophore and, therefore, the motion of protein residues around it.

To determine if our choice of isomerization reaction coordinate was reasonable, we compared our QM/MM split GFP calculations along the umbrella sampling *cis-trans* isomerization coordinate to the latest published reports on the different types of isomerization of HBDI, the GFP chromophore without a protein scaffold[12,13]. We assumed in this work that the chromophore in split GFP primarily undergoes a one bond flip (OBF) mechanism analogous to the most common isomerization mechanism in HBDI, where the chromophore twists about one central dihedral angle as it moves from *cis* to *trans* (Fig. 2d).[12,13] There are several possible mechanisms for the *cis* to *trans* isomerization to occur, including OBF about ϕ_I, OBF about ϕ_P and hula twist, among others. In all of these, once the chromophore is excited, the single and double bonds across the bridging carbons transition to a delocalized electronic structure, lengthening the double bond and creating flexibility in the bridge. As the different possible dihedral angles twist, and the energy gap between the ground and excited state decreases, the bridging carbon pyramidalizes with its hydrogen in the center of the bridge. At this point, the chromophore has reached a conical intersection, and can either relax back to its original *cis* form or to the *trans* form (Fig. 1c). It is well known that as the chromophore rotates along ϕ_I, the energy gap between the ground and excited state should decrease as the angle approaches 90° (from either the 0° or 180° directions).[12,13] Therefore, to validate our choice of OBF about ϕ_I to determine mutagenesis targets, we assumed that a reasonable umbrella sampling would approximate these features.

In our QM/MM optimizations based on the umbrella sampling, we observe twisted intramolecular charge transfer from the I-ring to the P-ring as ϕ_I approaches 90° (Fig.S2a). These

optimizations indicate a small region close to the conical intersection with a substantial difference in charge and geometry (ϕ_I=90-100), but minimal differences from ϕ_I=0-80 and 110-180 degrees (Supplementary Fig. 2b).

These findings are consistent with the known behavior of the chromophore, indicating that the structural sampling performed by umbrella sampling is sufficient for capturing these characteristics when analyzed at the QM/MM level despite the discrepancy in the charges on the chromophore close to the conical intersection. Supplementary Fig. 2c and d illustrates that these sampled structures capture this behavior on the ground and excited states, suggesting that the chromophore and its environment are behaving reasonably well during this sampling procedure.

S21. Umbrella sampling simulations

Because the protein environment surrounding the chromophore is asymmetric with respect to bond isomerization, we generated potential of mean force (PMF) profiles associated with clockwise and counterclockwise rotations along ϕ_I to determine the energy barrier to isomerization (Supplementary Fig. 4). We investigate both clockwise and counterclockwise rotation since the pocket around the chromophore is relatively open, and, as shown in Supplementary Fig. 4, there is not a large difference in energetic barrier to rotation in either direction despite the protein scaffold. The free energy barrier between the *cis* and *trans* configurations in the reference structure was ~25 kcal/mol (Supplementary Fig. 4), with the *trans* isomer ~15 kcal/mol above the *cis* isomer, and both energies significantly larger than the ~15 kcal/mol barrier and ~2 kcal/mol difference for the anionic chromophore in solution.[14] Because the chromophore is anchored to the protein, and a relatively rigid protein scaffold is more constricting than a mobile solvent environment, the large barrier associated with our PMF is reasonable. We recomputed the umbrella sampling pathways for the quadruple mutant IWI-A, and a theoretical AAA-A (Fig S4) mutant that includes the original AAA mutant plus the Asn121Ala mutation. AAA-A did not express measurable amounts and was only simulated. We found PMF barriers of around 21 kcal/mol in the positive direction and 26 kcal/mol in the negative direction for IWIA, and 22 kcal/mol in the positive direction/21 kcal/mol in the negative direction for AAA-A. Both indicate a substantial energetic lowering for the barrier to isomerization in the positive direction, supporting our hypothesis that the Asn121Ala mutation chosen from the umbrella sampling would have an effect on the isomerization. Higher level calculations are likely needed to move beyond a qualitative assessment of the excited state surface.

S21. Rotational correlation analysis and hydrogen bond occupancies

To compute the relationship of motion between the protein residues and the chromophore and determine possible mutagenesis targets, the center of mass of each residue was calculated and the correlation averaged over the umbrella sampling simulation windows with cpptraj. This generated a matrix of values where a positive value indicated a correlated motion. For example, each residue is exactly correlated with itself, and has a value of +1. A negative value indicates anticorrelation, i.e., residues that are moving in the opposite direction. A value close to zero indicates no correlation between the residue motion. We posited that residues that are strongly associated with the motion of the chromophore as it twists could be involved with the higher energetic barrier to isomerization in the protein as compared to free in solution. To narrow our list further, we then extracted the correlation vector associated with the chromophore relative to each other residue, i.e., if residues moved with, or out of the way, as the chromophore rotates (highly correlated or anticorrelated, respectively), and computed the difference as the chromophore moves from *cis* to *trans* (when ϕ_I =0-90° (Supplementary Fig. 3a) vs when ϕ_I =100-180° (Supplementary Fig. 3b)). This uncovers protein residues that are associated strongly with or against the motion of the chromophore, and that change their motion substantially as the chromophore moves from *cis* to *trans*. We combined this analysis with computed changes in hydrogen bonding occupancies over the course of the umbrella sampling to generate a list of potential mutagenesis targets that could affect the isomerization. Our chosen point mutation, Asn121, shows both a correlated motion with the chromophore and a competing transient hydrogen bond that forms during isomerization (present for ~20% of the isomerization pathway) (Supplementary Fig. 3c). The correlated motion is mitigated by the formation of this hydrogen bond—we therefore hypothesized that eliminating the hydrogen bond via point mutation would allow the isomerization to proceed more easily, since there would not be a competing hydrogen bond interaction that would interfere with this motion.

S22. Mutations of Asn121Ala

Although this work focuses on the structural features of strand-dissociation, we also predicted lowered *cis*-to-*trans* isomerization barriers for the Asn121Ala mutation. Although we expressed both AAA-A and IWI-A, only the expression of IWI-A yielded enough protein for further experiments. Although it displayed modestly lower fluorescence quantum yields, we did not observe faster dissociation or higher yields of the YFP product, compared with the corresponding triple mutant IWI. With a larger population of the *trans* complex, more strand-exchange is expected, although the rate should be similar to IWI if the strand-exchange step is still rate limiting and unaffected by the Asn121Ala mutation. However, the similar final YFP product concentrations of IWI and IWI-A indicate that the mutation affects chromophore rotation in both directions, thus also affecting the rate of thermal relaxation (Fig. 1c) back to the *cis* conformation in the ground state, resulting in relatively similar strand-exchange rates. Nonetheless, the observed FQYs in combination with the umbrella sampling simulations suggest that our predictions were qualitatively correct and are useful for predicting sites that can affect

the isomerization pathway. We therefore suggest that exploring the motion of surrounding residues as they relate to the twisting of the chromophore during isomerization indicate promising directions for future mutagenesis targets to improve *trans* product yield. Further mutations could be chosen in a similar fashion to improve isomerization yield for photoswitchable protein applications, i.e., mutating sites that show changes in correlated motion and hydrogen bonding as the chromophore moves from *cis* to *trans*. Apart from the Asn121Ala mutation, our simulations indicated that mutations at Thr205 and His148 could also affect isomerization rates. Since we have demonstrated the ability of reliably predicting useful mutation sites, these sites could be good candidates for future investigations of single sites that could affect both isomerization and strand-dissociation. In the particular case of split GFP, while isomerization is not the rate limiting step, we find that we were able to affect the FQY and thus potentially the isomerization yield in this way. That said, this is more broadly applicable to FP photoswitching in general rather than primarily split GFP strand dissociation. The motion of the dissociating strand is anticorrelated with the motion of the chromophore, meaning that as the chromophore twists, the dissociating strand is pushed away. This provides a possible link between the *cis* to *trans* isomerization motion, and the subsequent series of hydrogen bond changes that lead to eventual strand dissociation.

Supplementary Fig. 1 RMSD of replicated *cis* and *trans* simulations

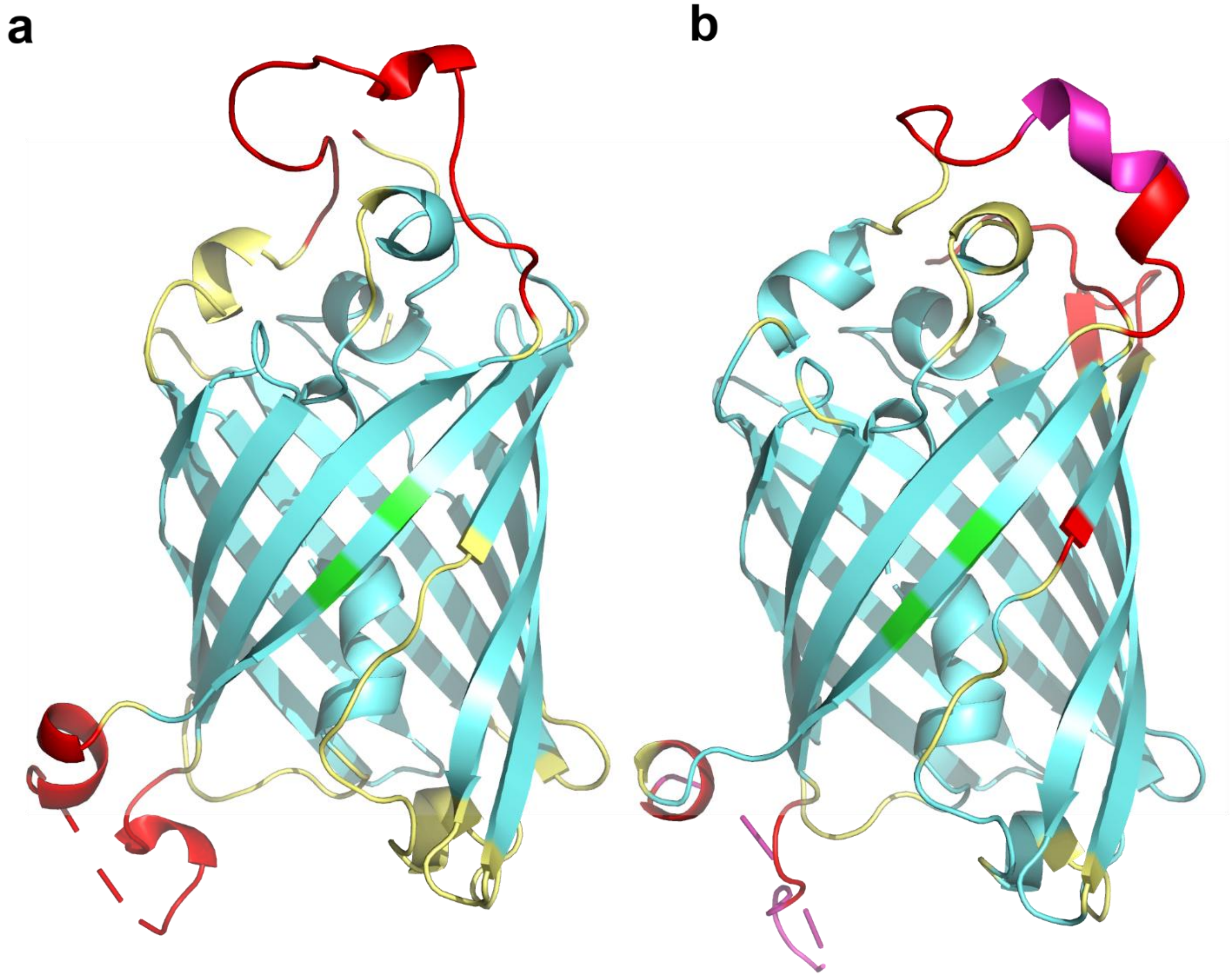

(a) *cis* and (b) trans cleaved GFPs after simulations at 300 K for 1μs, color-coded according to the RMSDs between replicates. Cyan: less than 1 Å. Yellow: 1 – 2 Å. Red: 2 – 15 Å. Magenta: 15 – 25 Å. The dotted lines indicate the cleaved loop (residues 45 – 73). Thr203 and Thr205 on strand 10 are shown in green for reference.

Supplementary Fig. 2 Electronic states

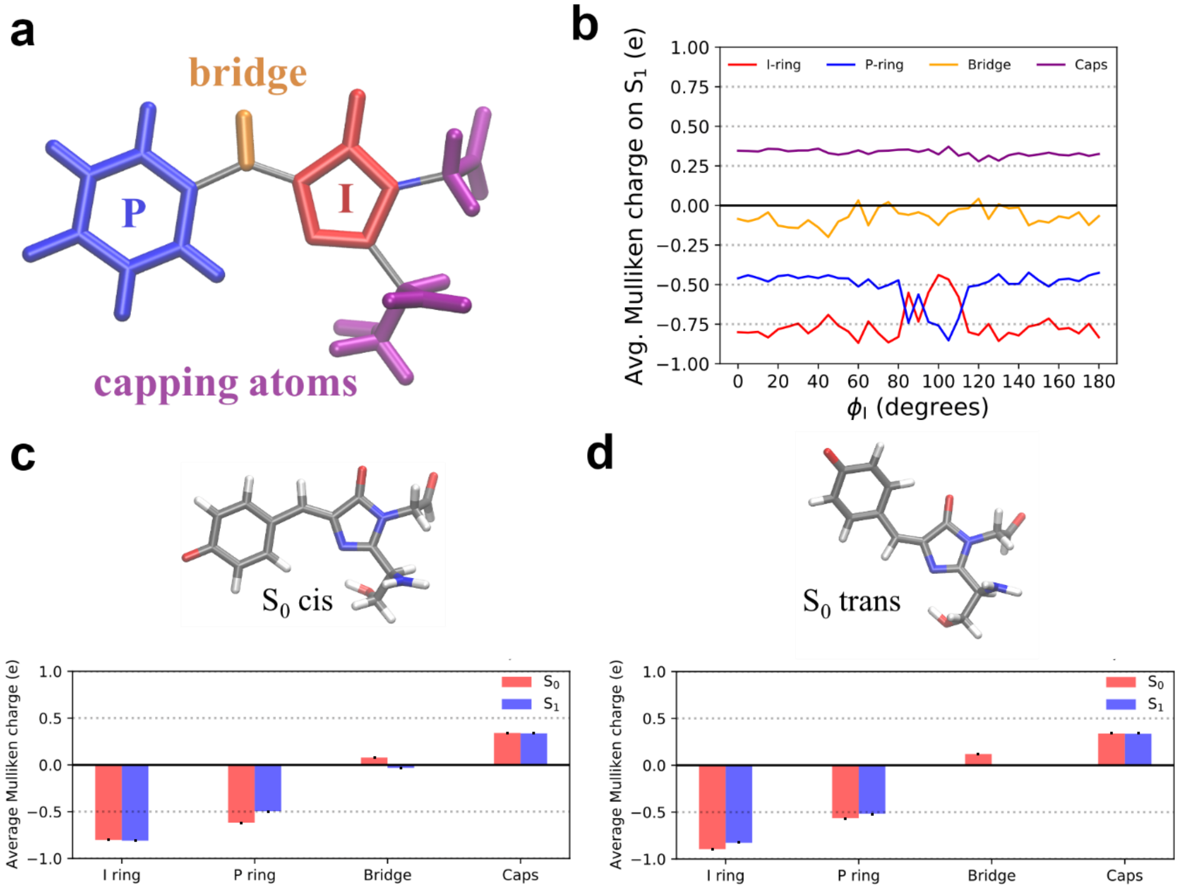

(a) *cis* chromophore colored by moiety: P-ring (blue), bridge (orange), I-ring (red). (b) Average Mulliken charge of atoms in each moiety as a function of ϕ_I. Structures were optimized on S_1 using the α(0.64)-SA3-CASSCF(4,3)/6-31G* level of theory (n=1 structure per 5°). Mulliken charges for moieties of the (c) *cis* and (d) *trans* forms of the GFP chromophore. Shown structures and charges correspond to S_0-optimized *cis* and *trans* configurations in the GFP protein scaffold (n=1 optimized *cis* structure and 1 optimized *trans* structure). Starting structures for *cis* and *trans* optimizations were obtained from ϕ_I=0° and ϕ_I=180° umbrella sampling windows, respectively. Average Mulliken charges were computed by averaging the atomic charges calculated across all atoms in their respective moiety. QM/MM optimizations were performed using the α(0.64)-SA3-CASSCF(4,3)/6-31G* level of theory.

Supplementary Fig. 3. Hydrogen bond networks in the *cis* and *trans* complexes

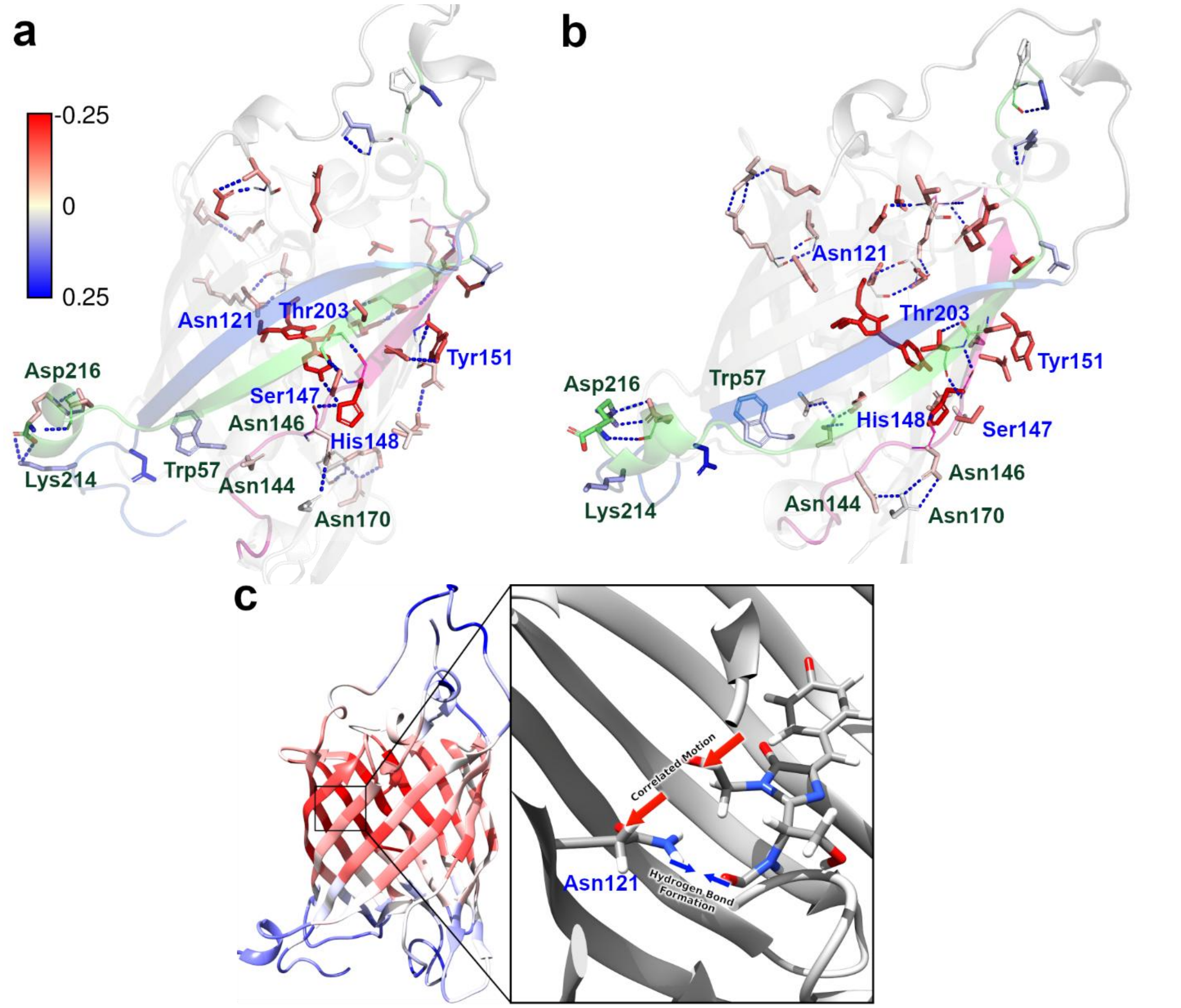

Summary of differences in hydrogen bonding and correlation of motion between the umbrella sampling from the chromophore $\phi_{I,,}$angle windows from 0-90° (top left), and from 90-170° (top right). Residues shown in stick are colored by their difference in correlation (blue, rotating with) or anticorrelation (red, rotating against) to the chromophore as the system moves from the midpoint of the isomerization to fully *trans*. Labeled residues are either directly associated with the chromophore (blue), or part of the allosteric network (green). The overall differences in correlated motion as the system moves from *cis* to *trans* is shown on the bottom left, mapped onto a representative protein structure. The specific mutation chosen from the blue (correlated) region, Asn 121, is shown as a zoom on the bottom right panel, indicating the direction of correlated motion in blue arrows and the competing transient hydrogen bond formation shown in red.

Supplementary Fig. 4. PMF plots

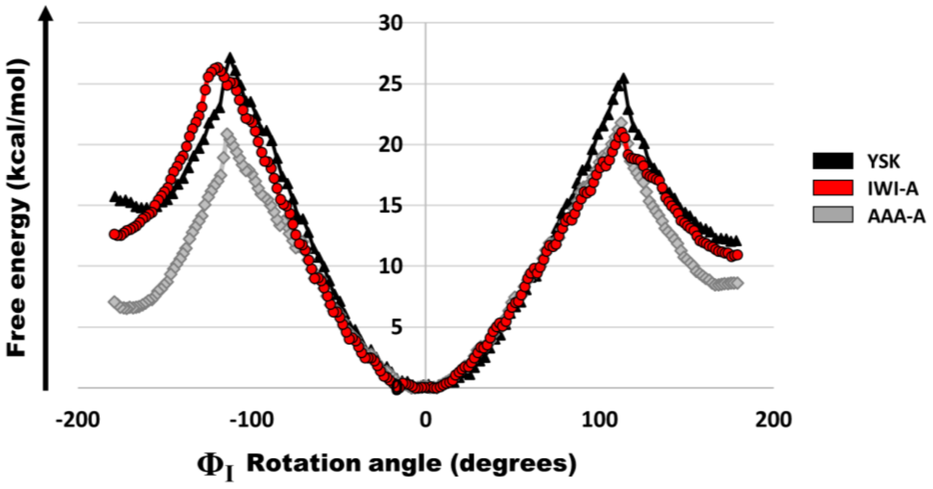

PMF of ground-state *cis*-to-*trans* isomerization of the chromophore in the reference structure YSK (black triangles), the expressed quadruple mutant IWI-A (red circles), and the AAA-A mutant (grey circles), along one-bond-flip coordinates, rotating ϕ_I (n=1 umbrella sampling per system). Errors computed by bootstrap analysis in WHAM via Monte Carlo (n=100 trials) in free energy range from 0.1 to 0.5 kcal/mol due to large force restraints.

Supplementary Fig. 5. Schematic topology of split GFP

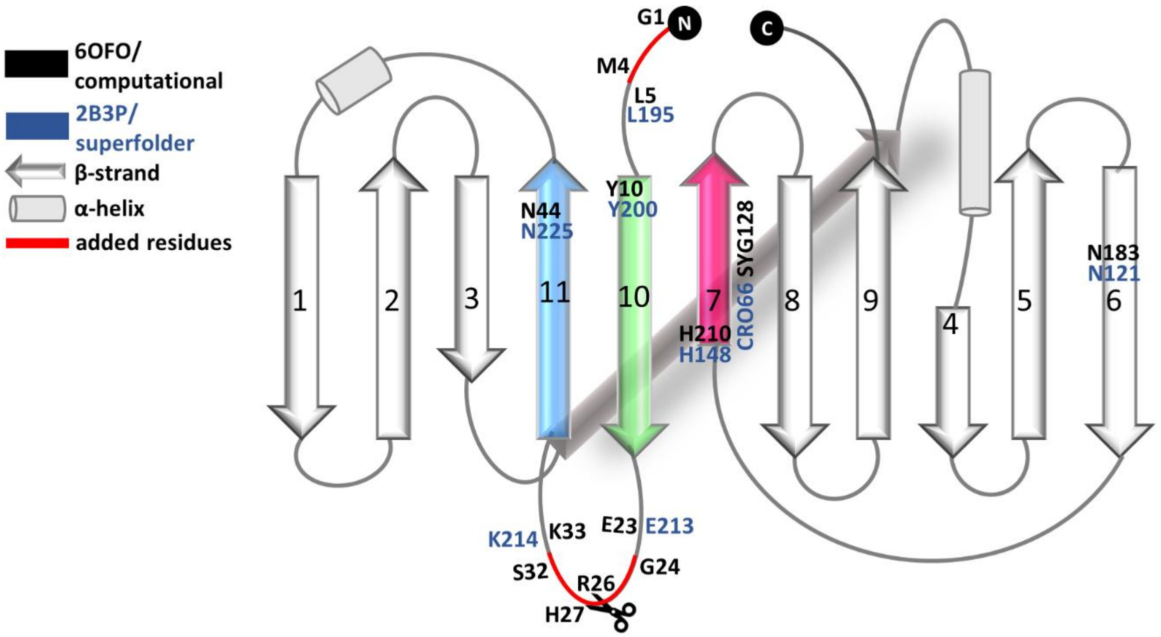

Schematic topology of split GFP, comparing the numbering of our computational model (and the 6OFO crystal structure) with the superfolder GFP (sfGFP) (PDB ID: 2B3P). Computational numbering is displayed in black while sfGFP numbering is shown in red (see Supplementary Table 3). Red lines indicate residues added in the sacrificial loop or due to circular permutation, which are absent in sfGFP. Circles indicate the N and C terminals. Strand numbers are indicated at the center of the strand. The internal helix is shown in gray. Strands 7 (pink), 10 (green), and 11 (blue) are highlighted. Scissors indicate the cleavage site.

Supplementary Fig. 6 Electronic state methods

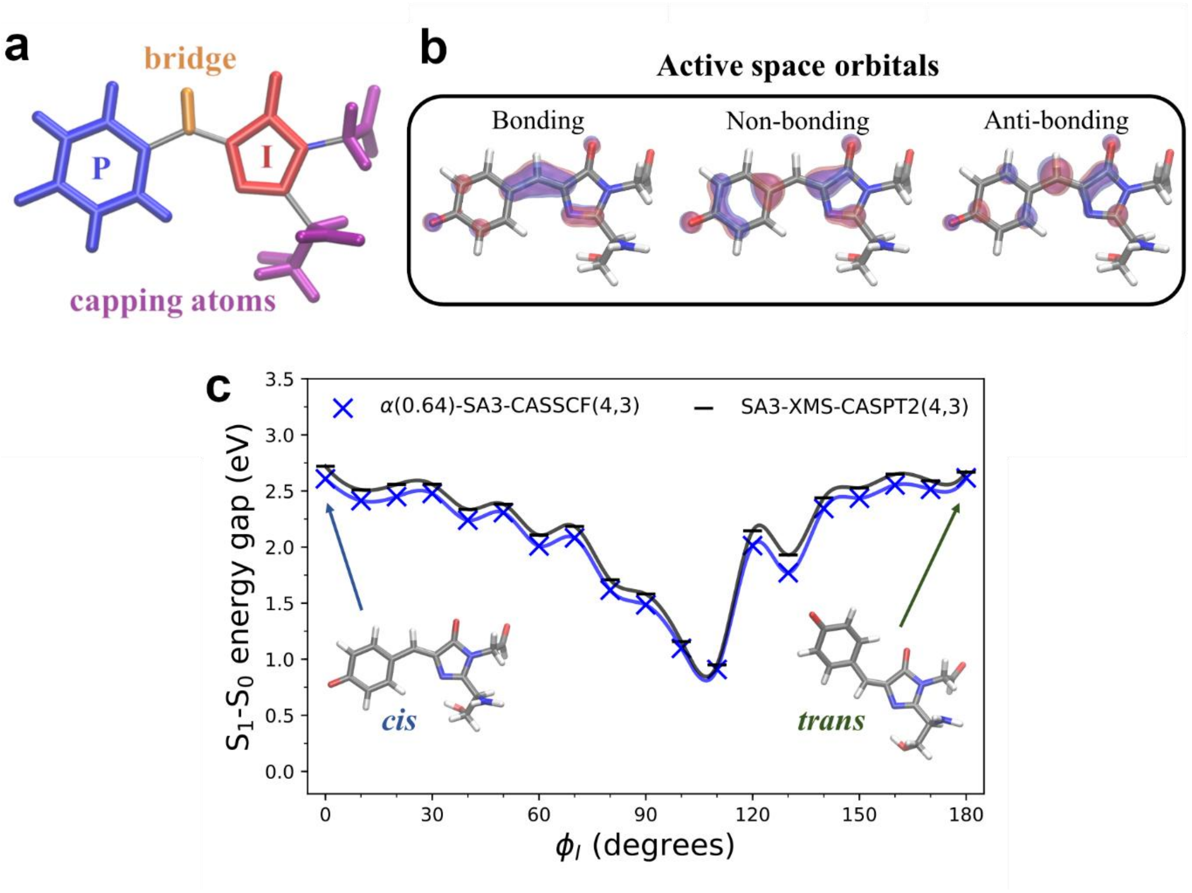

(a) *Cis* chromophore colored by moiety: P-ring (blue), bridge (orange), I-ring (red). (b) Active space orbitals of the GFP chromophore at the α(0.64)-SA3-CASSCF(4,3)/6-31G* level of theory. (c) Benchmark calculations for the energy gap between the S_0 and S_1 electronic states as a function of twisting along ϕ_I. Energies using the α(0.64)-SA3-CASSCF(4,3) method were obtained from QM/MM optimizations on S_0, and energies using the SA3-XMS-CASPT2(4,3) method were obtained from single point energy calculations on α-CASSCF optimized structures. The 6-31G* basis set was used for both methods. The depicted moieties of the anionic GFP chromophore are used for charge analysis as a function of ϕ_I twisting.

Supplementary Table 1. Strand exchange rates

Measured pseudo first order strand exchange rates ± standard deviation of replicates for laser-induced strand-exchange experiments using 488 nm laser irradiation at 26 mW in all experiments except the AAA (dark) where no external light source was used. Dark experiments were only done for AAA, as it was the fastest-photodissociating variant, and the best candidate for further exploration.

**Mutants**	**Rate ($10^{-4} \cdot s^{-1}$)**
YSK (Reference)	4 ± 2
ASK	9 ± 3
YAK	16 ± 1
YSA	3 ± 1
YSI	3 ± 1
YAA	14 ± 3
AAI	29 ± 1
AAA	85 ± 3
AAA (dark)	1 ± 1
LII	8 ± 1
LVI	8 ± 1
IWA	13 ± 3
IWI	10 ± 2
IWI-A	8 ± 1

Supplementary Table 2. Experimental fluorescence quantum yields

Protein	FQY [%]
YSK (Reference)	38 ± 1
AAA	40 ± 1
IWI	42 ± 1
IWI-A	34 ± 2

Supplementary Table 3. Conversion table between computational models and sfGFP crystal structure

Since the sequence of superfolder GFP (sfGFP) is the standard nomenclature in GFP research papers, we refer to amino acids according to their sfGFP numbering in our discussions. However, since there are differences between sfGFP and our complexes due to the circular permutation, placing the N-terminal on strand 10 and added residues due to the sacrificial loop, this conversion table can be used to convert between the computational models (corresponding to the 6OFO crystal structure sequence) and the sfGFP crystal structure (see amino acid sequence comparison on page 10 in this SI).

**strand 10**	**sfGFP**
Gly1, Ser2, His3, Met4	N/A
Leu5	195
Tyr10	200
Ser12	202
Thr13	203
Thr15	Ser205
Lys19	209
Glu23	213
Gly24, Thr25, Arg26	N/A
**strand 11**	**sfGFP**
His27, Ser28, Gly29, Ser30, Gly31, Ser32	N/A
Lys33	214
Arg34	215
Asp35	216
His36	217
His40	Leu221
Glu41	222
Asn44	225
**strand 7**	**sfGFP**
His 210	148
Asn211	149
Tyr213	151
**Other strands**	
Cys110	48
Asn183	121
**a-helix**	
GYS128	CRO66
Cys132	70

N/A: Not available (extra inserted loop in the 6OFO and computational structures)

Supplementary Table 4. Mass spectrometry

Expected mass was calculated as the average (av.) from the primary sequence using the online PeptideSynthetics Peptide Mass Calculator and reducing the mass to account for the maturation of the chromophore and the loss of the N-terminal methionine in the protein sequence (which is removed *in vivo*).

Mass of intact and cleaved GFP variants.

Mutants	Expected mass (Da)	Observed mass[(a)] (Da)
YSK (Reference)	30719	30730
ASK	30627	30638
YAK	30703	30716
YAA	30646	30657
IWI-A	30710	30722
AAA	30553	30565
AAI	30596	30607
IWA	30711	30723
IWI	30753	30764
LVI	30666	30694
LII	30680	30693
YSA	30662	28922[(b)]
YSI	30704	28966[(b)]
Truncated protein[(c)] of single, double, and triple mutants	25344	25356
Truncated quadruple mutant IWI-A	25301	25311

a) Proteins of ~30kDa have ± 15 Da deviations, depending on the protonation states.

b) Observed masses were consistent with the mass of the proteins, with the loss of the N-terminal HIS-tag

c) As all mutations were done on the strand that dissociates, the truncated protein after dissociation is the same for all single, double, and triple mutants.
