# Supplementary material for: Simulation-guided engineering of split GFPs with efficient β-strand photodissociation
Source: Nat Commun. 2023 Nov 16;14:7401. doi: 10.1038/s41467-023-42954-4 (PMC10654500; doi:10.1038/s41467-023-42954-4)
Supplement: Supplementary file 3 — Reporting Summary [file 41467_2023_42954_MOESM3_ESM.pdf]

nature portfolio

Corresponding author(s): Yasmin Shamsudin
Steven G. Boxer

Last updated by author(s): Sep 29, 2023

Reporting Summary

Nature Portfolio wishes to improve the reproducibility of the work that we publish. This form provides structure for consistency and transparency in reporting. For further information on Nature Portfolio policies, see our Editorial Policies and the Editorial Policy Checklist.

Statistics

For all statistical analyses, confirm that the following items are present in the figure legend, table legend, main text, or Methods section.

n/a	Confirmed	
☐	☒	The exact sample size (*n*) for each experimental group/condition, given as a discrete number and unit of measurement
☐	☒	A statement on whether measurements were taken from distinct samples or whether the same sample was measured repeatedly
☒	☐	The statistical test(s) used AND whether they are one- or two-sided<br>*Only common tests should be described solely by name; describe more complex techniques in the Methods section.*
☒	☐	A description of all covariates tested
☒	☐	A description of any assumptions or corrections, such as tests of normality and adjustment for multiple comparisons
☐	☒	A full description of the statistical parameters including central tendency (e.g. means) or other basic estimates (e.g. regression coefficient) AND variation (e.g. standard deviation) or associated estimates of uncertainty (e.g. confidence intervals)
☒	☐	For null hypothesis testing, the test statistic (e.g. *F*, *t*, *r*) with confidence intervals, effect sizes, degrees of freedom and *P* value noted<br>*Give P values as exact values whenever suitable.*
☒	☐	For Bayesian analysis, information on the choice of priors and Markov chain Monte Carlo settings
☒	☐	For hierarchical and complex designs, identification of the appropriate level for tests and full reporting of outcomes
☒	☐	Estimates of effect sizes (e.g. Cohen's *d*, Pearson's *r*), indicating how they were calculated

Our web collection on statistics for biologists contains articles on many of the points above.

Software and code

Policy information about availability of computer code

Data collection	The kinetic data were collected using PerkinElmer UV WinLab 6.4.0.973. Mass spectrometry data was collected with LC-MS using a Waters 2795 HPLC with ZQ single quadrupole MS. Fluorescence data were collected with a PerkinElmer LS 55 fluorescence spectrometer. The molecular modeling was done in Bioluminate 4.3. QM simulations was done using Gaussian16. Molecular dynamics simulations were done using AMBER 2018. QM/MM calculations were performed with the TeraChem 1.9.3/OpenMM7 interface.
Data analysis	VMD 1.9.3, pyMol2 for Windows and the AMBER2018 cpptraj module were used for analyzing structures and simulation trajectories. PMI-Intact 3.1-19 by Protein Metrics was used to analyze mass spectrometry data. Origin 2021b was used for statistical analysis.

For manuscripts utilizing custom algorithms or software that are central to the research but not yet described in published literature, software must be made available to editors and reviewers. We strongly encourage code deposition in a community repository (e.g. GitHub). See the Nature Portfolio guidelines for submitting code & software for further information.

Data

Policy information about availability of data

All manuscripts must include a data availability statement. This statement should provide the following information, where applicable:

- Accession codes, unique identifiers, or web links for publicly available datasets
- A description of any restrictions on data availability
- For clinical datasets or third party data, please ensure that the statement adheres to our policy

Files associated with performed simulations, including starting structures, parameters, simulation input files, and short videos of simulation trajectories, can be retrieved from the Zenodo server using the following URL: https://doi.org/10.5281/zenodo.7674800

Research involving human participants, their data, or biological material

Policy information about studies with human participants or human data. See also policy information about sex, gender (identity/presentation), and sexual orientation and race, ethnicity and racism.

Reporting on sex and gender	Not applicable
Reporting on race, ethnicity, or other socially relevant groupings	Not applicable
Population characteristics	Not applicable
Recruitment	Not applicable
Ethics oversight	Not applicable

Note that full information on the approval of the study protocol must also be provided in the manuscript.

Field-specific reporting

Please select the one below that is the best fit for your research. If you are not sure, read the appropriate sections before making your selection.

☒ Life sciences ☐ Behavioural & social sciences ☐ Ecological, evolutionary & environmental sciences

For a reference copy of the document with all sections, see nature.com/documents/nr-reporting-summary-flat.pdf

Life sciences study design

All studies must disclose on these points even when the disclosure is negative.

Sample size	Three strand-exchange experiments were conducted for each of the 12 engineered proteins, and for the reference protein.
Data exclusions	No data were excluded from the analyses.
Replication	All experimental measurements and unconstrained MD simulations were done in triplicates and successfully reproduced the results of this work.
Randomization	Different automatically generated random starting seeds were used for all molecular dynamics simulations. Randomization was not performed for fluorescence and kinetic experiments and are not applicable for those studies.
Blinding	Blinding was not performed for any simulations, kinetics, or fluorescence experiments and is not applicable for those studies.

Reporting for specific materials, systems and methods

We require information from authors about some types of materials, experimental systems and methods used in many studies. Here, indicate whether each material, system or method listed is relevant to your study. If you are not sure if a list item applies to your research, read the appropriate section before selecting a response.

Materials & experimental systems

n/a	Involved in the study
☒	☐ Antibodies
☒	☐ Eukaryotic cell lines
☒	☐ Palaeontology and archaeology
☒	☐ Animals and other organisms
☒	☐ Clinical data
☒	☐ Dual use research of concern
☒	☐ Plants

Methods

n/a	Involved in the study
☒	☐ ChIP-seq
☒	☐ Flow cytometry
☒	☐ MRI-based neuroimaging
